# First Uses of Chloroform and Ether in Buffalo1Read before the Medical Society of the County of Erie, January 14, 1896, on the occasion of its seventy-fifth anniversary.

**Published:** 1896-04

**Authors:** John Hauenstein

**Affiliations:** Buffalo, N. Y.; 309 Elmwood Avenue


### BUFFALO MEDICAL JOURNAL.

VOL. XXXV. APRIL, 1896. NO. 9.

### Original Communications.

### FIRST USES OF CHLOROFORM AND ETHER IN BUFFALO.[1]

BY JOHN HAUENSTEIN, M. D., Buffalo, N. Y.

IT MAY be because old men are generally considered garrulous and to have always something to say, that I have been charged by the committee of arrangements to give a brief account of the first uses of chloroform and ether in Buffalo and also of personal reminiscences of noted men of the profession at the commencement of my professional career. I will endeavor to discharge my trust as best I can.

The wonderful progress of the knowledge of medicine through the discoveries and introductions of new resources and means of combating disease, during the past half century, entitles this period to the appellation of the revolutionary era of the medical sciences. I will mention but a few of the more prominent subjects, such as auscultation and its branch, percussion, chloroform and ether, antisepsis, and, lastly, bacteriology. Besides, there have been added to the materia medica numerous valuable and potent medicines. My instructions, however, do not permit me to treat of all these subjects, yet I cannot forego the temptation of mentioning some of them, particularly auscultation when first practised in this city.

The great epoch, about the same time that I engaged in the practice of medicine, remarkable as a most important acquisition in diagnosis, particularly of heart and lung diseases, was that of the introduction of auscultation and its branch, percussion. Today it seems strange that such an important discovery should have been

1. Read before the Medical Society of the County of Erie, January 14, 1896, on the occasion of its seventy-fifth anniversary.

so long on its way to this country, for in 1819 already Laennec published his great work on auscultation, and yet when I attended my last term of lectures, in Geneva, in the winter of 1843 and 1844, no instructions had been thus far given on the subject. As an attempt, a negro was placed upon the platform, with chalk marks on his chest, intended to locate the different regions, the students being seated afar off, and having no opportunity given to apply their ears to the negro's chest.

When I returned, in January, 1844, from Geneva, there was not one physician in this city then who knew anything of the art of auscultation, unless he may have chanced to read some extracts from the *Journal de Médecine*, then edited by Laennec in Paris. Paris was then, and up to the time when Berlin succeeded to establish its claim, the center of medical science.

When Dr. George Burwell returned from Philadelphia, where he graduated and served a term as interne in the hospital, he was quite distinguished among the physicians here, having acquired some knowledge of auscultation and percussion. "George," as he was familiarly called, was frequently summoned by the different physicians to diagnosticate cases of heart and lung diseases which they were treating. From this we are to conclude that auscultation was beginning to be taught in some of the colleges of the larger seaboard cities. But I have been charged to say something more particularly about the first uses of chloroform and ether.

The next most important discovery came to us from over the Atlantic with more speed. In 1847, the introduction of chloroform as an anesthetic, by Dr. Simpson, of Edinburgh, marks one of the most important events in the history of medicine. If I were able to give you a pen picture of the scene that occurred in the operating room before the advent of chloroform, when an important operation was being performed, you would consider yourselves favored to be permitted to practise surgery after the introduction of anesthetics. The writhing and moaning of the patient, the almost convulsive efforts to release himself from the grasp of a half dozen assistants, was a scene that appealed to the strongest heart.

How different is the picture now presented! The patient lies calm and oblivious to all that is going on with and around him, occasionally making remarks as though he were enjoying pleasant company. Only once in a while we meet with an exceptional subject showing signs of a combative sort. After the operation and the discontinuance of the administration of the anesthetic, the patient does not remember having suffered any pain.

Of course, when a new remedial agent makes its first appearance, it is experimented with in all kinds of cases, and such was the way with me. Soon after chloroform came to be used as an anesthetic I was called to a patient suffering from tetanus, caused by a wound near the knee-joint, inflicted with an adze. The young man was perfectly rigid. Two men, one taking hold of the back part of his head, the other of his heels, could carry him from one bed to another like a rail. I kept the patient more or less under the influence of chloroform for a number of days and he got well. I was afterward reproached by a number of the then older physicians for not having shown them the case. I have since regretted my neglect, but I innocently considered the use of chloroform then, in such cases, as a matter of course.

That no pain whatever is experienced during an operation, when under the influence of chloroform, I can testify from personal experience. About twenty years ago, in the city of New York, when returning from the Centennial Exposition in Philadelphia, I was entertained by friends, and during the enjoyment of dinner my hernia, with which I had been troubled for many years, became strangulated. I was taken back to my hotel and Dr. Carnochan, my friend's family physician, was summoned, and everything was attempted to reduce it, but to no purpose. Next day Prof. Hamilton was called in consultation. Both physicians tried their best, but without success. Having had some experience myself in strangulated hernias and being in the hands of two eminent physicians, Dr. Carnochan having been a pupil of Sir Ashley Cooper, who was an expert in hernial operations, I concluded that the sooner the operation of releasing the hernia was performed the better the result would be. So I informed the doctors that I was ready and the operation was performed. It was a femoral hernia, and a portion of omentum protruded and was adherent in the canal, which during the operation was ligated and subsequently served an excellent purpose as a prop, becoming the means of a radical cure. I was under the influence of chloroform two hours, but never felt the least pain.

Dr. George Burwell made use of chloroform in every case of obstetrics he attended, for many years, but in later years he abandoned its general use. According to my experience it predisposes to post-partum hemorrhage, on account of its relaxing property. Chloroform was used almost exclusively as an anesthetic in this city up to recent times, and, as far as I know, with general satisfaction. Dr. Winne had me, for many years, administer chloroform in almost every operation he performed.

Dr. Morton, of Boston, administered ether in the Massachusetts General Hospital, October 16, 1846, on the occasion of a surgical operation performed by Dr. J. C. Warren. He had, before, made several successful experiments in the extraction of teeth. He procured a patent under the name of Lithean, but he had a hard road to travel. About the time his claim underwent an elaborate investigation in congress, he visited this city for the purpose of interesting the profession in his case. The honor of the discovery was awarded to him by a select committee of congress, but he never realised any pécuniary comfort from the government.

Of late years, ether has, to a great extent, superseded chloroform, because of the number of fatal cases which was attributed to the latter. I regret this change and believe that, in some such fatal cases, carelessness was the cause, or that such subjects had heart trouble. I have administered chloroform very many times and never witnessed any serious results. It is said that the medical records of the Crimean war do not furnish one fatal case attributable to chloroform.

I am inclined to believe that with proper care and more gradual administration, with plenty of air and a due regard to the conditions of the heart, but very few cases would result in death. It ought also to be remembered that ether requires a much longer time to produce narcosis. Many subjects do not take as kindly to it as they do to chloroform. The fighting and apparent suffering of many, in the first stage of its administration, makes an observer inclined to the belief that the patient is not much benefited by the anesthetic, ether.

Anesthesia was waiting for the arrival of a partner for a long time ; at last it came, and is now known by the name of antisepsis. Lister, another Scotchman, introduced the agent to the profession. Both anesthesia and antisepsis are so closely related that the one cannot be mentioned by the surgeon without speaking of the other. Together they have revolutionised the practice of surgery. No such important and daring operations as are now daily performed were undertaken before the two agents mentioned were at the command of the surgeon. Operations that were formerly considered very dangerous and hazardous, terminating frequently in death, are now made with excellent results.

Tait, the successful gynecologist of England, is said to be an exception in not using antiseptics. If this be so, the rule is confirmed, since there is no rule without an exception.

There is yet another contributor to the advancement of medicine, whose name I mentioned in the beginning of this paper, but of whom I will say but a few words. Bacteriology is beginning to loom up on this side of the Atlantic. It was but an infant a few years ago, but already is accorded an important place in the science of medicine. Malignant anthrax, hydrophobia, diphtheria and, possibly, tetanus, that were considered monsters of diseases, have become manageable as a consequence of the lessons taught by bacteriology. Its value as a combatant of disease is established and it challenges the imagination to contemplate its future.

As regards the other part of my charge, on which it is expected I should say something—namely, of personal reminiscences of noted men of the profession in Buffalo, at an early period, it cannot be expected, in consequence of the limited time allotted to me for the different subjects, that I can say much about the noted men of fifty or more years ago. My remarks, therefore, will be brief.

Fifty-two years ago, from which time my remarks commence, Dr. Josiah Trowbridge was the oldest of the noted men of the profession then practising. He was an able physician, who kept well abreast of the advancement of the medical science by strict application to its study. He was once mayor of the city.

Dr. Flint was at the head of the doctors then in Buffalo. He gave public lectures, repeatedly, on the subject of anatomy, illustrating the different parts and organs on a manikin. As a consultant he was a model. He left this city for New York about the same time that Prof. Hamilton did. Both became professors in the Bellevue Medical College and both, as you all know, became distinguished and famous in their particular branches of the art of medicine.

Dr. James P. White acted a prominent part among the professional men as a manager and leader. He excelled in administrative qualities ; he was a manager, as I will illustrate. In a meeting of this society, January, 1881, Dr. White came to me and said, " You ought to be our next president." I had never thought, nor had I any aspiration after the office, but the little vanity that possessed me made me reply that if he thought I was the proper person, I would accept the office if elected. In less than fifteen minutes after, he had manipulated the members and I was afterward elected president. When I attended my last course of lectures in the Geneva Medical College, Dr. White came there to carry out his scheme of having the college removed to this city, or, at least, the prominent members of its faculty, such as Professors Hamilton, Webster and Hadley. He succeeded as regards the professors. But as almost all of you knew him personally, with, perhaps, few exceptions, and remember him, it is not necessary for me to say anything further about his superior attainments and distinguished characteristics.

Dr. Charles Winne was considered one of our best surgeons, if not the best, before Professor Hamilton came to this city and again after the latter left for the city of New York to assume the professorship of surgery in the Bellevue Hospital Medical College. Dr. Winne had a large general practice and devoted every leisure moment to the study of subjects of medicine.

I must not forget my preceptor, Dr. F. L. Harris, who, with a collegiate education, was well informed on the subjects of his profession, and would, therefore, have had a brilliant future here, but left this city for New York, where he practised for many years, until he died.

Dr. Sprague, a prominent practitioner, with a very large general practice, was also popular as a surgeon. Dr. Loomis was in partnership with Dr. Sprague, and performed some nice surgical operations and had a large practice.

Dr. Pratt was somewhat brusque in manners and speech, but of a kindly disposition ; his practice was among older families. After practising for years he went to Paris to study up some particular branch of medicine that he was interested in.

Dr. Barnes was a fine gentleman and possessed a collegiate education. He pursued his considerable practice unostentatiously afoot until in his later years he got himself a horse and chaise, but in consequence of an accident whilst driving he gave up his vehicle and ever afterward returned to pedestrianism.

Dr. Bryant Burwell, the father of George, had a considerable practice, and was also noted among his colleagues.

Dr. W. K. Scott, who spoke German, was president of this society in 1844. His practice was limited, devoting some time to municipal affairs as city surveyor and engineer.

There may have been a few others, then practising medicine, and belonging to the regular profession, who might deserve mention, but my acquaintance with them and recollection go no further than the above referred to.

Some differences of opinion and views of purposes manifested themselves about the time the Buffalo Medical College was to be inaugurated. Two factions came on the field, led, the one by Dr. White, the other by Dr. Winne. But it is not in good taste to perpetuate the memory of the apple of discord by relating the particulars of the strife—it is enough for my purpose to indicate vaguely the cause in the few words of Shakespeare: "To be, or not to be."

About twenty years ago, Professor Hamilton and myself, both involved in the above questions of unpleasantness, but on opposite sides, met on an occasion, and there and then canceled our differences, embraced each other and parted as friends.

In conclusion, I heartily wish you all a happy and ripe old age, and if the progress of our art keeps apace in the future with the past seventy-five years, some of you will witness such brilliant achievements as are now beyond our comprehension.

309 Elmwood Avenue.

